# An Electric Fish-Based Arithmetic Optimization Algorithm for Feature Selection

**DOI:** 10.3390/e23091189

**Published:** 2021-09-09

**Authors:** Rehab Ali Ibrahim, Laith Abualigah, Ahmed A. Ewees, Mohammed A. A. Al-qaness, Dalia Yousri, Samah Alshathri, Mohamed Abd Elaziz

**Affiliations:** 1 Department of Mathematics, Faculty of Science, Zagazig University, Zagazig 44519, Egypt; rehab100r@yahoo.com (R.A.I.); abd_el_aziz_m@yahoo.com (M.A.E.); 2 Faculty of Computer Sciences and Informatics, Amman Arab University, Amman 11953, Jordan; Aligah.2020@gmail.com; 3 Department of Computer, Damietta University, Damietta 34517, Egypt; ewees@du.edu.eg; 4 State Key Laboratory for Information Engineering in Surveying, Mapping and Remote Sensing, Wuhan University, Wuhan 430079, China; alqaness@whu.edu.cn; 5 Electrical Engineering Department, Faculty of Engineering, Fayoum University, Fayoum 63514, Egypt; day01@fayoum.edu.eg; 6 Department of Information Technology, College of Computer and Information Sciences, Princess Nourah bint Abdulrahman University, Riyadh 84428, Saudi Arabia; 7 Artificial Intelligence Research Center (AIRC), Ajman University, Ajman 346, United Arab Emirates

**Keywords:** swarm models, feature selection (FS), metaheuristic (MH), electric fish optimization (EFO), arithmetic optimization algorithm (AOA)

## Abstract

With the widespread use of intelligent information systems, a massive amount of data with lots of irrelevant, noisy, and redundant features are collected; moreover, many features should be handled. Therefore, introducing an efficient feature selection (FS) approach becomes a challenging aim. In the recent decade, various artificial methods and swarm models inspired by biological and social systems have been proposed to solve different problems, including FS. Thus, in this paper, an innovative approach is proposed based on a hybrid integration between two intelligent algorithms, Electric fish optimization (EFO) and the arithmetic optimization algorithm (AOA), to boost the exploration stage of EFO to process the high dimensional FS problems with a remarkable convergence speed. The proposed EFOAOA is examined with eighteen datasets for different real-life applications. The EFOAOA results are compared with a set of recent state-of-the-art optimizers using a set of statistical metrics and the Friedman test. The comparisons show the positive impact of integrating the AOA operator in the EFO, as the proposed EFOAOA can identify the most important features with high accuracy and efficiency. Compared to the other FS methods whereas, it got the lowest features number and the highest accuracy in 50% and 67% of the datasets, respectively.

## 1. Introduction

The huge increase of data volume results in different challenges and problems such as irrelevant, high dimensionality, and noisy data [1]. Therefore, such problems affect the efficiency and accuracy of the machine learning algorithms and lead to high computational costs. Feature selection (FS) approaches have been utilized to reduce computational costs and to boost classification accuracy [2]. FS methods are generally used to capture data properties by selecting a subset of relevant features [3]. Additionally, they removed unnecessary and noisy data [3]. FS methods have been widely employed in different fields, such as human action detection [4], text classification [5], COVID-19 CT images classification [6], neuromuscular disorders [7], data analytics problems [8], parameter estimation of biochemical systems [9], MR image segmentation [10,11], and other applications [12,13,14].

There are three types of FS approaches, called wrapper, filter, and embedded [15]. Wrapper-based methods use the learning technique to assess the selected features, whereas filter-based methods use the properties of the datasets. Embedded-based methods learn which features best contribute to the accuracy of the classification model while it is being created. Therefore, filter-based methods are more efficient and faster than wrapper-based methods. The optimal FS method can be considered as a method that can minimize the number of selected features and maximize the accuracy of the classifier [15].

Metaheuristic (MH) optimization algorithms have been widely adopted to address complex optimization tasks, including FS. In literature, several MH algorithms have been used successfully to solve FS problems, such as particle swarm optimization (PSO) [16], differential evolution (DE) [17], genetic algorithms (GA) [18], as grasshopper optimization algorithm (GOA) [19], gravitational search algorithm (GSA) [20], slime mould algorithm (SMA) [3], Harris hawk optimization (HHO) algorithm [21], marine predators algorithm (MPA) [22], Aquila optimizer [23] and others [8,9,10,11,23].

According to the NFL theorem, no one algorithm can solve all problems. Thus, the hybridization concept is widely adopted to address several complex problems, including FS. Following the hybridization concept, we propose a new FS approach using a new variant of the electric fish optimization (EFO). The EFO is a recently proposed MH algorithm inspired by the natural behavior of the nocturnal electric fish [24]. It was evaluated in different and complex optimization problems, and it showed significant performance, except in high dimensional problems due to its slow convergences and getting stuck in local minima. Thus, we use a new optimizer, called the arithmetic optimization algorithm (AOA), to overcome the shortcomings of the traditional EFO algorithm. The AOA is inspired by mathematical operations and proposed by Abualigah et al. [25]. It has been adopted in several applications, such as proton exchange membrane fuel cells by extreme learning machine [26], multilevel thresholding segmentation [27], real-world multiobjective problem [28], and damage assessment in FGM composite plates [29].

The proposed algorithm, EFOAOA, works by splitting the tested dataset into training and testing sets, which represent 70% and 30% of all the data, respectively. Then we set the initial value for a set of individuals that represents the solutions for the FS problem. To assess these individuals, their Boolean versions are computed, and the fitness value is computed based on the features corresponding to Boolean ones. The next process is to determine which individual has the best fitness value and using it as the best individual. Then the operators of AOA and EFO are competitive in the exploration phase to discover the feasible region which contains the optimal solutions. This leads to an increase in the convergence towards the optimal solution. The operators of traditional EFO within the exploitation phase are used. The process of enhancing the value of individuals is conducted until reached the stop criteria. Then the testing set is reduced by using only the features that correspond to ones in the binary version of the best individual, and the performance is computed using different measures. To the best of our knowledge, this is the first time using either EFO or its modified version for feature selection problems.

Our main objectives and contributions can be summarized as follows:

- Propose a new modified version of electric fish optimization using the operators of arithmetic optimization algorithm to enhance exploration ability.
- Apply the enhanced EFOAOA as an alternative FS technique to remove the irrelevant features, which leads to improve the classification efficiency and accuracy.
- Use eighteen UCI datasets to assess the efficiency of the developed EFOAOA and compared it with well-known FS methods.

The rest of this paper is organized as follows: Section 2 introduces the similar FS method from previous literature. Section 3 presents the background of electric fish optimization and arithmetic optimization algorithm. In Section 4, the stages of the developed method are presented. Section 5 presents the experimental results and their discussions. Finally, the conclusion and future works are given in Section 6.

## 2. Related Works

In this section, we highlight a number of MH algorithms that were developed for feature selection problems. Chaudhuri and Sahu [30] proposed a modified version of the crow search algorithm (CSA) for FS. They used time-varying flight length for balancing the search process (exploration and exploitation). They evaluated eight variants of the developed FS method, and they tested them with 20 UCI datasets benchmark datasets, and it showed prominent performance.

A hybrid FS method based on binary butterfly optimization algorithm (BOA) and information theory was proposed by [31]. The developed method called information gain binary BOA (IG-bBOA) overcomes the shortcomings of the traditional BOA algorithm, and it achieved exceptional performance compared to several MH algorithms.

Maleki et al. [32] used the genetic algorithm as a feature selection method to improve the lung cancer disease classification process. A k-nearest-neighbors classifier was adopted to classify the stage of patients’ disease. The evaluation outcomes confirmed that GA improved classification accuracy.

Song et al. [33] introduced an FS approach based on a new variant of the PSO algorithm, called bare bones PSO. The main idea is to use a swarm initialization technique depending on label correlation. Also, two operators called supplementary, and deletion operators are used to enhance the exploitation process. More so, to avoid getting stuck in local minima, they developed an adaptive flip mutation operator. It was employed with kNN for several datasets, and it was compared to several MH algorithms to verify its performance.

In [34], the authors presented FS approach, called GWORS, using a combination between grey wolf optimizer (GWO) and rough set for mammogram image analysis. The GWORS was compared to well-known FS methods, and it obtained competitive performance. Tubishat et al. [35] developed an FS based on a dynamic salp swarm algorithm (SSA). The SSA was developed based on two methods. The first method is developed to update salps’ position, where the second one is to improve the local search process of the traditional SSA. The developed SSA was applied with kNN classifier, evaluated with well-known benchmark datasets, and compared to the traditional SSA and several MH algorithms.

Dhiman et al. [36] proposed a binary emperor penguin optimizer (EPO) for FS. They used twenty-five datasets to evaluate this approach with extensive comparisons to the state-of-art methods. Overall, the binary version of the EPO showed superior performance compared to the original one. In [21], an FS method based on a hybrid HHO algorithm and simulated annealing was proposed. In [37], the genetic algorithm was used with Elastic Net for feature selection. Neggaz et al. [38] presented an FS approach using the Henry gas solubility optimization (HGSO) algorithm. Ewees et al. [3] proposed an FS technique using a hybrid of slime mould algorithm and firefly algorithm. It was evaluated with different datasets, including two high dimensions QSAR datasets. 

Yousri et al. [6] developed a new FS method to enhance COVID-19 CT images classification based on an improved cuckoo search (CS) optimization algorithm. The fractional-order (FO) calculus and heavy-tailed distributions are used to enhance the performance of the traditional CS algorithm. In general, many MH algorithms, including hybrid methods, have been developed for various FS applications, and they showed good performance compared to traditional methods, as described in [39,40].

## 3. Background

### 3.1. Electric Fish Optimization

Electric fish optimization (EFO) is proposed in [1], which is inspired by the emergence of several optimization techniques. The electric fish solutions (N) are initialized randomly by the search space, considering the area’s boundaries:

(1) $$x_{ij}=x_{min\;j+rand\;\left(x_{max\;j}-x_{min\;j}\right)}$$

where $x_{ij}$ is the position number j in the solution number I, max, and min are the maximum and minimum boundaries, respectively.

In the EFO, as in nature, positions with a larger frequency use effective electrolocation, and others utilize passive electrolocation. The frequency value is given between the maximum and minimum of the fitness function values:

(2) $$f_{i}^{t}=f_{min}+\left(\frac{fit_{worst}^{t}-fitt_{i}^{t}}{fit_{worst}^{t}-fitt_{best}^{t}}\right)\left(f_{max}-f_{min}\right)$$

where $f_{i}^{t}$ is the fitness value of the solution number i at iteration number t. $fit_{worst}^{t}\;\mathrm{and}\;fitt_{best}^{t}$ are the worst and best obtained fitness functions values. $f_{max}-f_{min}$ are the max and min fitness functions values.

The amplitude cost of the solution number I ($A_{i}$) is determined as follows:

(3) $$A_{i}^{t}=\alpha A_{i}^{t-1}+\left(1-\alpha \right)f_{i}^{t}$$

where α is a value in range [0, 1].

#### 3.1.1. Active Electrolocation

The active range estimation is calculated as follows:

(4) $$r_{i}=\left(x_{\max j}-x_{\min j}\right)A_{i}$$

To discover neighboring solutions in the available space, estimate the distance is required between the current solution and other solutions. The distance between the solution number *i* and *k* is calculated as follows:

(5) $$d_{ik}=\left|\left|x_{i}-x_{j}\right|\right|=\sqrt{\overset{d}{\underset{j=1}{\sum }}{\left(x_{ij}-x_{kj}\right)}^{2}}$$

If at least one neighbor is found in the active space, Equation (6) is used; otherwise, Equation (7) is used:

(6) $$x_{ij}^{cand}=x_{ij}+\varphi \;\left(x_{kj}-x_{ij}\right)$$

(7) $$x_{ij}^{cand}=x_{ij}+\varphi r_{i}$$

where k is a random selected solution, φ is a value between [−1, 1], $x_{ij}^{cand}$ is the candidate positions of the solution number *i*.

#### 3.1.2. Passive Electrolocation

The probability of the solution number i in an active space is determined as follows:

(8) Pk=Akdik∑j ∈ NAAjdij

Using different approaches, such as roulette wheel selection, *K* solutions are determined from *N_A_* using Equation (8). *A* source location ($x_{rj}$) is defined using Equation (9). The new positions are then produced using Equation (10):

(9) $$x_{rj}=\frac{\sum_{k=1}^{K}A_{k}x_{kj}}{\sum_{k=1}^{K}A_{k}}$$

(10) $$x_{ij}^{new}=x_{ij}+\varphi \;\left(x_{rj}-x_{ij}\right)$$

Although it is unusual, there might be a situation where a solution with a higher rate gives passive electrolocation. To evade this, Equation (11) is determined to choose the parameter values:

(11) $$x_{ij}^{cand}=\{\begin{array}{c} x_{ij}^{new}\;\;\;\;\;\;\;rand>f_{i}\;\\ x_{ij}\;\;\;\;\;\;\;\;\;\;\;\;\;\;otherwise \end{array}$$

The final action of passive space is to change one parameter of the solution number *i* by Equation (12) to improve the likelihood of a trait denoting exchanged:

(12) $$x_{ij}^{cand}=x_{min\;j+rand\;\left(x_{max\;j}-x_{min\;j}\right)}$$

If the value of the parameter number *j* of the solution number *i* oversteps the boundaries, it is relocated to the following limitations:

(13) $$x_{ij}^{cand}=\{\begin{array}{c} x_{min\;j}\;\;\;\;\;\;\;\;\;\;\;\;\;\;\;\;\;\;\;\;\;\;\;\;\;\;\;x_{ij}^{cand}<x_{min\;j}\\ x_{ij}^{cand}\;\;\;\;\;\;\;\;x_{max\;j}>x_{ij}^{cand}>x_{min\;j}\\ x_{max\;j}\;\;\;\;\;\;\;\;\;\;\;\;\;\;\;\;\;\;\;\;\;\;\;\;\;\;\;x_{ij}^{cand}>x_{max\;j} \end{array}$$

### 3.2. Arithmetic Optimization Algorithm

The arithmetic optimization algorithm (AOA) is an optimization depends on using arthimentical operations [2]. The improvement process starts with choosing the search mechanisms based on Equation (14):

(14) $$MOA\left(t\right)=Min+t\times \left(\frac{Max-Min}{T}\right)$$

where *t* is the active iteration, which is in range [1, T]. *Min* and *Max* are the smallest and highest values for this function. The mathematical of the search mechanisms is given as follows.

#### 3.2.1. Exploration Part

The exploration process is given in Equation (3). This search is performed when rand > *MOA*, rand is a random number, and *MOA* can be found in Equation (14). The D search is executed when *rand* < 0.5; otherwise, the M search will be executed:

(15) $$x_{i,j}\left(t+1\right)=\{\begin{array}{c} best\left(x_{j}\right)÷\left(MOP+\epsilon \right)\times \left(\left(UB_{j}-LB_{j}\right)\times \mu +LB_{j}\right),rand<0.5\\ best\left(x_{j}\right)\times MOP\times \left(\left(UB_{j}-LB_{j}\right)\times \mu +LB_{j}\right),\;\;otherwise\; \end{array}$$

(16) $$MOP\left(t\right)=1-\frac{t^{\left(\frac{1}{\alpha }\right)}}{T^{\left(\frac{1}{\alpha }\right)}}$$

where $x_{i}\left(t+1\right)$ is the solution number i at the iteration number *t*, $x_{i}$, *j*(*t*) is the position number *j* in the solution number *i*, and best ($x_{j}$) is the best solution yet. *µ* and *α* are parameter values fixed to 0.5, 5, respectively [3]. *t* is the used iteration, and *T* is the total used iterations.

#### 3.2.2. Exploitation Part

This search section is executed when *rand* ≤ *MOA*. The S search is executed when *rand* < 0.5; otherwise, the A search will be executed. Thus, the exploitation search, based on S and A, typically averts the local search problem. The following mathematical presentation is used to express the exploration search mechanisms:

(17) $$x_{i,j}\left(C\_Iter+1\right)=\{\begin{array}{c} best\left(x_{j}\right)-MOP\times \left(\left(UB_{j}-LB_{j}\right)\times \mu +LB_{j}\right),\;r3<0.5\\ best\left(x_{j}\right)+MOP\times \left(\left(UB_{j}-LB_{j}\right)\times \mu +LB_{j}\right),otherwise\; \end{array}$$

To summarize, the processes in the AOA begin with stochastic solutions formed over some constraints. By the development rule, the search tools attempt to obtain the optimal solution with possible conditions. The primary practice in improving the worked solutions is the strategy of the best global solution. A transition approach (named *MOA*) is employed to preserve the stability among the search mechanisms using a linear function rose in the range [0.2, 0.9]. The exploration tools are practiced when *rand* > *MOA*; otherwise, the exploitation tools will be used. In searching sections, the operators will be practiced randomly. Eventually, the AOA is stopped by touching the end criterion.

## 4. Proposed FS Method

**Figure 1:**
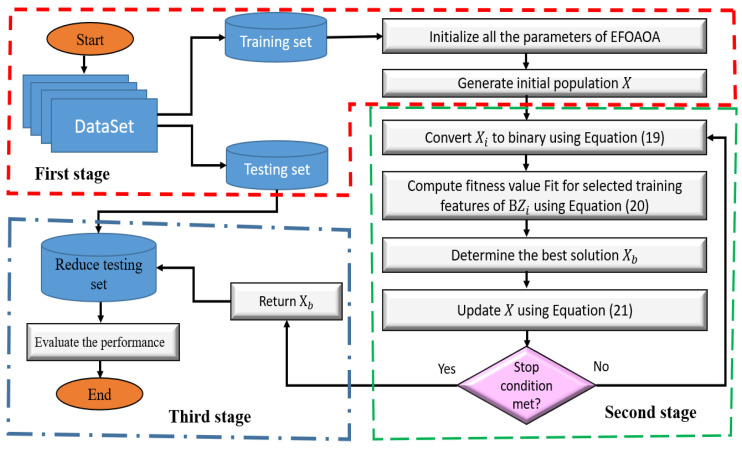
The different stages of the EFOAOA method as FS approach. First stage contains the dataset. Second stage contains the updating process. Third stage contains the evaluation process.

The framework of the developed FS technique depends on improving the effienciy of EFO using the operators of AOA is given in Figure 1.

The main target of using AOA is to enhance the exploration ability of EFO since it has the largest influence on its ability to discover the feasible region that contains the optimal solutions. The proposed FS approach, named EFOAOA, begins with dividing the data into training and testing sets, which represent 70% and 30%, respectively. Then the random values for N individuals are assigned, and for each of them, the fitness value is computed. Then the individual that has the best fitness value is used as the best individual. After this process, the updating of the solution is performed using the operators of EFO in the exploitation phase, while during the exploration phase, either the operators of AOA or traditional EFO are used according to random probability. The process of updating individuals is performed again until the stop conditions are reached. Thereafter, the testing set is reduced according to the best individual and the performance of the developed EFOAOA as FS is evaluated using different metrics. The details of the EFOAOA are given in the following paragraphs.

### 4.1. First Stage

At this stage, the initial individuals are generated, which represents the population of solutions. The formulation of this process is given as:

(18) $$X_{i,j}=\left(UB_{j}-LB_{j}\right)\times rand+UB_{j}-LB_{j},\;i=1,2,\ldots ,N,\;\;\;\;j=1,2,\ldots ,D$$

where $UB_{j}$ and $LB_{j}$ are the upper and lower boundary at jth dimension. N represents the total number of individuals and D is the dimension of each solution, and it represents the total number of features. rand∈[0,1] is a random number. 

### 4.2. Second Stage

The main aim of this part of the developed EFOAOA is to update the individuals until they reached to the stop conditions. This is achieved through a set of steps; the first step is to convert each individual $X_{i}\;$ into a binary individual using the following equation:

(19) $$BX_{i,j}=\{\begin{array}{c} 1\;\;\;\;\;if\;X_{i,j}\geq 0.5\\ 0\;\;\;otherwise \end{array}$$

The next step is to use the training features that corresponding to ones in $BX_{i,j}$ to learn the KNN classifier and compute the fitness value that is defined as:

(20) $$Fit_{i}=\alpha \times \gamma +\left(1-\alpha \right)\times \left(\frac{\left|BX_{i,j}\right|}{D}\right)$$

In Equation (20), γ is the error classification using KNN classifier and $\left|BX_{i,j}\right|$ is the total number of ones (i.e., relevant features). α∈[0,1] is the factor that balances between two parts of fitness value. The main reason of using KNN due to its simplicity and efficiency, as well as, it has one parameter. In addition, it has been provided better performance than most of other classifiers in different applications. Since, it stores the data of the training set and this.

The step after that is to allocate the best individual $X_{b}$ that has the smallest $Fit_{b}$. Then compute the frequency ($f_{i}$) and amplitude ($A_{i})$ for each $X_{i}$ using Equations (2) and (3), respectively. According to the value of $f_{i}$ the individuals will be updated using either the active phase (i.e., $f_{i}>rand$) or passive phase (i.e., $f_{i}\leq rand$). During the active phase, the operators of traditional EFO are used to update the individuals as given in Equations (4)–(7). Meanwhile, inside the passive phase, the operators of AOA and EFO are competitive to improve the individuals, and this is conducted according to the following formula:

(21) $$X_{i,j}=\{\begin{array}{c} Eq\left(8\right)-Eq\left(13\right)\;\;\;\;\;if\;Pro\geq 0.5\\ Eq\left(14\right)-Eq\left(17\right)\;\;\;otherwise \end{array}$$

where Pro∈[0,1] refers to probability of using either AOA (i.e., Equations (14)–(17)) or EFO (i.e., Equations (8)–(13)) to update $X_{i,j}$. In case the update $X_{i,j}$ has fitness value better than its old value, then update $X_{i,j}$ is used; otherwise, the old one is kept. Then the stop conditions are checked in case they are satisfied then the best individual $X_{b}$ is returned from this stage.

### 4.3. Third Stage

In this stage, the testing set is reduced by selecting only the features that corresponding ones in the binary version of $X_{b}$. Then applied reduced testing set is applied to the trained classifier (KNN) and predicts the output of the testing set. The next process is to evaluate the quality of the output using different metrics. The setps of EFOAOA are given in Algorithm 1.
**Algorithm 1. Steps of EFOAOA**1. Input: the dataset which has D features, number of individuals (N), number of iterations (tmax), and parameters of EFOAOA **First Stage** 2. Split data into twp parts (i.e., training and testing) 3. Construct the population X using Equation (18). **Second Stage** 4. t=1 5. While (t<tmax) 6. Convert each $X_{i}$ into its binary version using Equation (19). 7. Compure fitness value for each $X_{i}$ based on training set as in Equation (20). 8. Find the best individual $X_{b}$. 9. Update X using Equation (21). 10. t=t+1 11. EndWhie **Third Stage** 12. Reduce the testing set based on selected features from $X_{b}$. 13. Evalaute the performance using different measures

### 4.4. Complexity of EFOAOA

The time complexity of EFOAOA depends on the complexity of EFO and AOA. Since, time complexity of EFO and AOA are given in Equations (22) and (23), respectively:

(22) $$O\left(\mathrm{EFO}\right)=\{\begin{array}{c} O\left(tmax\times N\times D\right)\;\;\;in\;best\;case\\ \left(tmax\times N^{2}\times D\right)\;\;\;\;in\;worst\;case \end{array}$$

(23) O(AOA)=O(tmax×N×D)    

So, the complexity of EFOAOA can be represented as:

(24) $$O\left(\mathrm{EFOAOA}\right)=\{\begin{array}{c} O\left(K_{p}\left(tmax\times N\times D\right)+\left(1-K_{p}\right)\left(tmax\times N\times D\right)\right)\;\;\;in\;best\;case\\ O(K_{p}\left(tmax\times N^{2}\times D\right)+\left(1-K_{p}\right)\left(tmax\times N\times D\right))\;\;\;\;in\;worst\;case \end{array}$$

(25) $$O\left(\mathrm{EFOAOA}\right)=\{\begin{array}{c} O\left(tmax\times N\times D\right)\;\;\;\;\;\;\;\;\;\;\;\;\;\;\;\;\;\;\;\;\;\;\;\;\;\;\;\;\;\;\;\;\;in\;best\;case\\ O(\left(tmax\times N\times D\right)\left(K_{p}\left(N+1\right)+1\right)\;\;\;\;in\;worst\;case \end{array}$$

where $K_{p}$ stand for the number of solutions updated using operators of EFO.

## 5. Experimental Results

This section evaluates the performance of the developed EOFAOA method over eighteen benchmark datasets. In addition, we compare the proposed EOFAOA with ten FS algorithms.

### 5.1. Dataset Description and Parameter Setting

The description of eighteen UCI datasets is listed in Table 1. From this table, it can be observed that these datasets are collected from different real-life applications, and they have different characteristics. For example, different numbers of samples, features, and classes. Moreover, the developed EFOAOA is compared with namely EFO, AOA, MRFO, bGWO, HGSO, MPA, TLBO, SGA, WOA, and SSA. The parameter of each algorithm is assigned based on its original work. The common parameters between these methods are the number of iterations and number of individuals, and we put their values to 50 and 20, respectively. In addition, each of these methods is conducted 25 times to make the comparison fair between them. The comparison bead on six measures: the average, worst (MAX), best (MIN), standard deviation (Std) of the fitness value, and accuracy (Acc).

### 5.2. Results and Discussion

**Figure 2:**
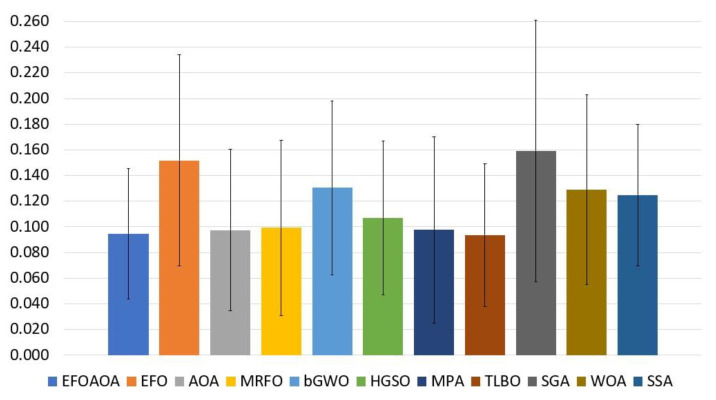
Average of fitness value over all the tested datasets.

Table 2 lists the feature selection results of all methods using the average of the fitness function values. From this table, we can notice that the proposed EOFAOA got the better average in eight out of 18 datasets (i.e., Breastcancer, D2, D4, D6, D8, D10, D12, and D17), and it was ranked first. Whereas the AOA method achieved the second rank with three out of 18 datasets (i.e., D3, D9, and D18), followed by TILBO and MRFO with two datasets for each one, but the average of the TILBO in all datasets was better than MRFO. The SGA recorded the worst results at all. Figure 2 illustrates the performance of the EFOAOA based on the average results of the fitness functions values. In addition, the developed EFOAOA is more stable than other FS methods in terms of fitness value as can be seen from the bars of standard deviation.

The results of the standard division of the fitness function for all methods are recorded in Table 3. The developed EOFAOA approache showed good stability in all datasets compared to the other approaches, and it obtained the lowest Std value in four out of 18 datasets (i.e., D1, D5, D8, and D17), followed by TILBO and EOF with the lowest std values in three datasets for each one whereas, both MRFO and MPA also showed good stability. The WOA recorded the worst in this measure.

The best results of the fitness values are recorded in Table 4. From this table, we can see that the proposed EOFAOA showed competitive results with TILBO method. The EOFAOA obtained the best results in three datasets (i.e., D3, D4, and D15), whereas the TILBO reached the minimum average in four datasets (i.e., D7, D12, D16, and D17), and they showed the same Min results in the D10 dataset. The worst method was the EFO.

Regarding the worst results of the fitness values, as in Table 5, the proposed EOFAOA is superior to other compared methods and achieved the best results in 39% of all datasets (i.e., D4, D5, D6, D9, D11, D13, and D15), and it showed competitive results in the rest of the datasets. The AOA achieved the second rank by obtaining the best results in 28% of the dataset (i.e., D3, D8, D10, D17, and D18), followed by TILBO and MPA with 22% for each one. The rest methods were arranged in the following sequence the MFO, HGSO, SSA, EFO, bGWO, and WOA; whereas, the SGA showed the worst results.

Furthermore, the best number of the selected features is recorded in Table 6. In this measure, the algorithms should try to select the most relative features meanwhile achieving the highest accuracy values. As shown in Table 6, the EOFAOA got the lowest features number in 50% of the datasets. The WOA obtained the second rank and got the lowest features number in 44% of the datasets, followed by MRFO, bGWO, MPA, HGSO, TILBO, AOA, SSA, and EFO; whereas, the SGA recorded the largest features number among all methods.

**Figure 3:**
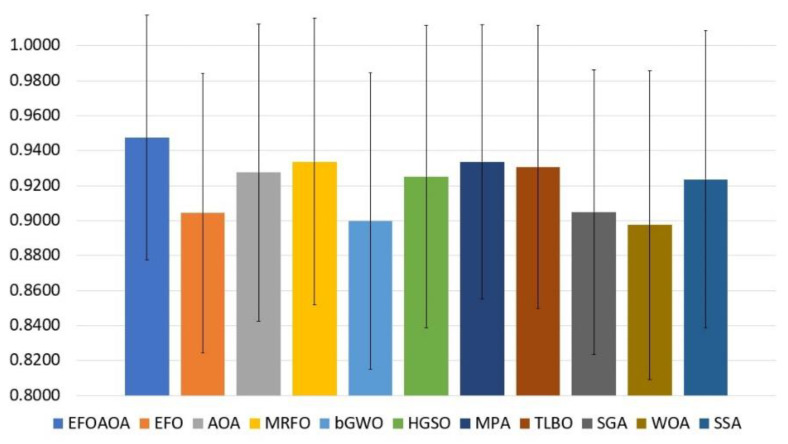
Average of accuracy measure overall the tested datasets.

The results of the proposed EOFAOA and all the compared methods in terms of accuracy are recorded in Table 7. The results of this measure show that the proposed EOFAOA is superior to other compared approaches. It achieved the highest accuracy in 39% of all datasets and got the same accuracy with the other methods in 28% of all datasets. This result indicates that the EOFAOA is able to select the most relative feature and save the quality of the classification accuracy. The second rank was recorded by MRFO followed by AOA, MPA, TILBO, SSA, HGSO, EFO, and bGWO whereas, the WOA showed the lowest accuracy in all datasets. Figure 3 indicates that the proposed EFOAOA got the highest accuracy in the average of all datasets, as well as, it is considered more stable than other methods as can be observed from standard deviation bars.

Moreover, for further analysis of the results of the developed methods, the Friedman test is used. This test is a non-parametric test that provides a statistical value that indicates if there is a significant difference between the developed method and other methods. Table 8 shows the Friedman rank test results of the compared methods based on the accuracy, the number of selected features, and fitness value results. In this table, the EFOAOA obtained the first rank, followed by MPA, MRFO, AOA, TLBO, and SSA. In terms of the average of fitness value, the developed EFOAOA allocates the second rank following HGSO. In addition, the developed EFOAOA provides the best mean rank in terms of the number of selected features.

### 5.3. Comparison with Other FS Techniques

In this part, the results of developed EFOAOA are compared with well-known FS approaches that depends on MH technqiues. These FS approaches including the two whale optimization algorithms [41,42], binary bat algorithm (BBA) [43], enhanced GWO (EGWO) [44], BGOA [45,46], PSO, biogeography-based optimization (BBO), two binary GWO algorithms, namely bGWO1 and bGWO2 [47], AGWO [44], satin bird optimizer (SBO) [44], and enhanced crow search algorithm (ECSA) [48].

Table 9 illustrates the results of classification accuracy of the developed EFOAOA and other methods. From these results it can be seen that the developed EFOAOA has high ability of improve the classification accuracy overall the tested datasets except D5, D7, D14, where PSO, bGWO2, and BGOA are the best, respectively. This incdicate the high ability of EFOAOA to select the relevant features with persevring the accuracy of classification. 

To sum up, the previous results show that there is an obvious enhancement in solving feature selection problems when using the proposed EOFAOA method. The EOF is improved extremely by using the operators of the AOA in its structure. Therefore, the EOFAOA can be considered as an efficient and effective optimization algorithm for solving feature selection problems.

## 6. Conclusions and Future Work

Aiming for proposing an efficient feature selection (FS) optimizer, this paper proposed an innovative variant of the electric fish algorithm (EFO) via integrating the operator of the arithmetic optimization algorithm (AOA) into the EFO ‘s exploration phase. The EFO has a drawback while handling large-dimensional optimization problems for solving that a hybrid variant named EFOAOA was proposed. The proposed EFOAOA was applied on eighteen different real-life datasets to tackle the FS optimization task. The EFOAOA was compared with the basic EFO, AOA and MRFO, bGWO, HGSO, MPA, TLBO, SCA, WOA, and SSA through a set of statistical metrics, namely the average value, worst, best, the standard deviation of the fitness function, and the classification accuracy as well as the Friedman test as a non-parametric test. The comparisons revealed that the EFOAOA got the best average fitness in eight out of 18 datasets, the lowest Std value in four out of 18, and the lowest features number in 50% of the datasets. Accordingly, the proposed EFOAOA got the highest accuracy in the average of all datasets; this result indicated that the EOFAOA was able to select the most relative feature and saved the quality of the classification accuracy at the same time. Also, the operators of the AOA play an essential role in improving the exploration stage of the original EFO algorithm.

For future work, the proposed EFOAOA will be examined with several applications include image segmentation, parameter estimation, and time-series forecasting. Also, we will add more improvement to the EFOAOA stages by using different techniques such as chaotic maps and opposition-based learning.

## Figures and Tables

**Table 1:** Description of datasets.

Datasets	Number of Instances	Number of Classes	Number of Features	Data Category
Breastcancer (D1)	699	2	9	Biology
BreastEW (D2)	569	2	30	Biology
CongressEW (D3)	435	2	16	Politics
Exactly (D4)	1000	2	13	Biology
Exactly2 (D5)	1000	2	13	Biology
HeartEW (D6)	270	2	13	Biology
IonosphereEW (D7)	351	2	34	Electromagnetic
KrvskpEW (D8)	3196	2	36	Game
Lymphography (D9)	148	2	18	Biology
M-of-n (D10)	1000	2	13	Biology
PenglungEW (D11)	73	2	325	Biology
SonarEW (D12)	208	2	60	Biology
SpectEW (D13)	267	2	22	Biology
tic-tac-toe (D14)	958	2	9	Game
Vote (D15)	300	2	16	Politics
WaveformEW (D16)	5000	3	40	Physics
WaterEW (D17)	178	3	13	Chemistry
Zoo (D18)	101	6	16	Artificial

**Table 2:** Average of fitness values for all methods.

	EFOAOA	EFO	AOA	MRFO	bGWO	HGSO	MPA	TLBO	SGA	WOA	SSA
D1	**0.05086**	0.08232	0.06313	0.06836	0.06789	0.06006	0.07245	0.05554	0.10176	0.07381	0.05515
D2	**0.03246**	0.08842	0.03979	0.04558	0.08085	0.09111	0.04004	0.06375	0.12798	0.06994	0.07771
D3	0.04276	0.09892	**0.02707**	0.04760	0.10753	0.03019	0.03773	0.03728	0.10184	0.07382	0.08947
D4	**0.04258**	0.07928	0.04769	0.05393	0.14146	0.08515	0.05013	0.04667	0.19231	0.15975	0.10079
D5	0.20119	0.31339	0.24958	0.21919	**0.19977**	0.29019	0.26147	0.23568	0.33061	0.21696	0.27428
D6	**0.12821**	0.16615	0.12897	0.16470	0.20376	0.13009	0.12966	0.14573	0.19581	0.21598	0.14504
D7	0.03926	0.14565	0.04345	0.05246	0.08166	0.10571	0.08089	**0.03523**	0.12058	0.09927	0.08312
D8	**0.05747**	0.09921	0.06224	0.07450	0.09547	0.09491	0.06559	0.07837	0.11478	0.09712	0.09473
D9	0.12232	0.22421	**0.07972**	0.13178	0.15640	0.10091	0.09194	0.09377	0.18178	0.12868	0.08353
D10	**0.03777**	0.08082	0.04769	0.05116	0.09975	0.07679	0.04974	0.04911	0.11787	0.11761	0.09988
D11	0.06720	0.20062	0.16158	**0.01497**	0.04886	0.02392	0.07389	0.05461	0.20224	0.04076	0.05703
D12	**0.04757**	0.13738	0.09371	0.08346	0.09649	0.08322	0.07768	0.05713	0.08905	0.06727	0.13175
D13	0.18818	0.23455	0.14667	0.15566	0.23525	**0.11152**	0.14556	0.15677	0.20475	0.23364	0.22838
D14	0.21972	0.24198	0.20556	0.23154	0.25477	0.22279	**0.19815**	0.21479	0.22787	0.25719	0.24612
D15	0.05950	0.10425	0.04325	**0.03783**	0.05325	0.04650	0.06358	0.05683	0.10517	0.04567	0.08233
D16	0.29012	0.31154	0.27168	0.27501	0.30259	0.29423	0.26385	**0.25680**	0.30750	0.29956	0.30268
D17	**0.02385**	0.07962	0.03385	0.03949	0.05705	0.04218	0.03846	0.02667	0.08782	0.06987	0.05077
D18	0.05375	0.04250	**0.01000**	0.03917	0.06595	0.03875	0.01667	0.01958	0.05625	0.05327	0.04292

**Table 3:** Standard division of fitness values for all methods.

	EFOAOA	EFO	AOA	MRFO	bGWO	HGSO	MPA	TLBO	SGA	WOA	SSA
D1	**0.00124**	0.00366	0.00131	0.00664	0.00613	0.00181	0.00130	0.00447	0.01204	0.01129	0.00528
D2	0.00865	0.01054	0.01133	0.00678	0.01138	**0.00581**	0.00678	0.00870	0.00665	0.01152	0.00805
D3	0.00106	0.01378	0.00293	0.00872	0.01959	0.00187	0.00621	**0.00000**	0.01187	0.01534	0.00872
D4	0.01714	0.02816	0.00344	0.00651	0.08102	0.03297	0.00772	**0.00199**	0.08227	0.09634	0.04179
D5	**0.00000**	0.00949	0.01551	**0.00000**	0.00487	0.04174	0.02358	0.01158	0.01642	0.02620	0.01642
D6	0.02298	0.03400	0.01463	0.02765	0.03192	0.01825	0.01521	0.02023	**0.01045**	0.02253	0.02399
D7	0.01878	**0.00326**	0.01858	0.01648	0.00944	0.01646	0.01097	0.00793	0.00910	0.01774	0.01740
D8	**0.00403**	0.01087	0.00742	0.00498	0.01414	0.00795	0.00796	0.00765	0.01060	0.01500	0.00921
D9	0.02278	0.00942	0.00977	**0.00749**	0.02549	0.01341	0.02158	0.02974	0.02710	0.05968	0.01315
D10	0.03748	0.02079	**0.00344**	0.00689	0.04160	0.02357	0.00397	0.00693	0.04253	0.04101	0.03841
D11	0.03584	**0.00069**	0.05446	0.00753	0.02659	0.01160	0.01485	0.03567	0.00234	0.03144	0.00253
D12	0.02743	0.01133	0.01301	0.01392	0.02020	0.01696	0.01644	0.01843	**0.01060**	0.01282	0.01681
D13	0.01394	0.01378	0.00761	0.02229	0.01772	0.01516	0.00969	0.01633	0.01193	**0.00716**	0.01581
D14	0.01232	0.01193	**0.00000**	0.00090	0.01766	0.00929	0.00061	0.00345	0.02312	0.01796	0.01883
D15	0.03054	0.01055	0.00873	0.00730	0.02007	**0.00529**	0.00704	0.01465	0.02316	0.01677	0.01729
D16	0.00862	**0.00769**	0.01420	0.01110	0.01149	0.01028	0.01350	0.01212	0.00855	0.01740	0.01023
D17	**0.00000**	0.01723	0.00421	0.01002	0.01171	0.00966	0.00581	0.01450	0.01607	0.01386	0.00910
D18	0.00342	0.01118	0.00839	0.01168	0.01567	0.00350	0.00386	**0.00220**	0.00747	0.01187	0.00521

**Table 4:** Best fitness values results for all methods.

	EFOAOA	EFO	AOA	MRFO	bGWO	HGSO	MPA	TLBO	SGA	WOA	SSA
D1	0.06373	0.07659	0.06254	0.06548	0.05730	0.05905	0.07190	0.05262	0.08302	0.05905	**0.04619**
D2	0.05158	0.07825	0.03123	0.03246	0.06070	0.07860	**0.02912**	0.04579	0.11070	0.04912	0.06456
D3	**0.02159**	0.07909	0.02500	0.03944	0.06638	0.02909	0.03125	0.03728	0.07694	0.05603	0.07694
D4	**0.03385**	0.05385	0.04615	0.04615	0.04615	0.05385	0.04615	0.04615	0.06154	0.04615	0.05385
D5	0.20119	0.29685	0.22877	0.21919	**0.19669**	0.24169	0.23719	0.23269	0.30323	0.21019	0.25054
D6	0.13846	0.12821	0.12179	0.12308	0.16282	**0.10641**	0.11282	0.12051	0.16923	0.17308	0.11282
D7	0.07423	0.14076	**0.01765**	0.02738	0.06744	0.06541	0.06156	**0.01765**	0.10476	0.07423	0.04412
D8	0.07984	0.08635	0.05292	0.06424	0.06832	0.08094	**0.05028**	0.06455	0.10031	0.06595	0.08217
D9	0.08659	0.21628	0.06992	0.11556	0.10651	0.06239	0.06992	0.05556	0.13222	0.04711	**0.04444**
D10	**0.04615**	0.06604	**0.04615**	**0.04615**	0.05385	0.05385	**0.04615**	**0.04615**	0.06154	0.06154	0.05385
D11	0.00400	0.19969	0.09746	0.00523	0.02031	0.01077	0.02215	0.00708	0.19815	**0.00308**	0.05323
D12	0.05643	0.12619	0.07619	0.05976	0.06476	0.05643	0.04667	**0.03000**	0.07500	0.04810	0.10452
D13	0.17121	0.22121	0.13485	0.09848	0.19394	**0.08939**	0.12424	0.13939	0.17727	0.21818	0.20455
D14	0.21024	0.22431	0.20556	0.23073	0.23073	0.21788	**0.19792**	0.21198	0.21198	0.23542	0.22899
D15	**0.02350**	0.09500	0.03375	0.02500	0.03375	0.03750	0.05500	0.02750	0.06250	0.03625	0.05250
D16	0.28010	0.30310	0.25740	0.24820	0.28470	0.27580	0.23800	**0.23540**	0.29510	0.27300	0.28430
D17	0.05385	0.06346	0.03077	0.02308	0.03846	0.03077	0.03077	**0.01538**	0.06923	0.04615	0.03846
D18	0.05000	0.02500	**0.00625**	0.02500	0.04375	0.03125	0.01250	0.01875	0.04375	0.03750	0.03125

**Table 5:** Worst fitness values results for all methods.

	EFOAOA	EFO	AOA	MRFO	bGWO	HGSO	MPA	TLBO	SGA	WOA	SSA
D1	0.07659	0.08595	0.06548	0.08944	0.08183	**0.06373**	0.07659	0.06548	0.12278	0.09762	**0.06373**
D2	0.07316	0.10491	0.05702	0.06035	0.10105	0.10228	**0.05368**	0.08193	0.13649	0.08561	0.08825
D3	0.04353	0.11638	**0.03125**	0.07694	0.14310	0.03319	0.04784	0.03728	0.13297	0.10366	0.10797
D4	**0.05304**	0.11423	0.05385	0.06604	0.23719	0.15473	0.07504	0.05385	0.30962	0.28219	0.17273
D5	**0.20119**	0.31992	0.27042	0.21919	0.21208	0.33342	0.29496	0.27754	0.35592	0.31165	0.30004
D6	**0.14974**	0.21026	0.15513	0.21154	0.26923	0.15769	0.15513	0.17308	0.20897	0.25128	0.20256
D7	0.11611	0.14959	0.06653	0.08509	0.09959	0.12991	0.10750	**0.04888**	0.13894	0.12788	0.10568
D8	0.10179	0.11286	**0.06990**	0.08214	0.12151	0.10505	0.07545	0.08941	0.13403	0.11599	0.10889
D9	**0.09156**	0.23889	0.09215	0.14556	0.20516	0.11984	0.12889	0.12540	0.22778	0.24667	0.10222
D10	0.13992	0.10973	**0.05385**	0.07054	0.17854	0.12192	**0.05385**	0.07054	0.21062	0.18362	0.18681
D11	**0.03077**	0.20154	0.24838	0.03385	0.09046	0.05415	0.08246	0.13477	0.20646	0.08523	0.06185
D12	0.12595	0.15262	0.11262	0.10762	0.12429	0.11095	0.10286	**0.08619**	0.10810	0.08667	0.16214
D13	**0.13061**	0.25606	0.15303	0.17121	0.26515	0.14394	0.16515	0.19394	0.22273	0.23788	0.25909
D14	0.24010	0.25590	0.20556	0.23247	0.29236	0.24184	**0.19965**	0.22135	0.27934	0.29167	0.29635
D15	**0.04250**	0.12250	0.05750	0.05000	0.09750	0.05500	0.07625	0.07375	0.14375	0.09500	0.11000
D16	0.29960	0.32100	0.29540	0.29420	0.32150	0.31520	0.28820	**0.27410**	0.32150	0.32290	0.31570
D17	0.05385	0.10192	**0.03846**	0.05385	0.07115	0.05385	0.04615	0.05385	0.11923	0.08654	0.06923
D18	0.05625	0.05000	**0.02500**	0.05625	0.08661	0.04375	**0.02500**	**0.02500**	0.06875	0.08036	0.05000

**Table 6:** Selected features numbers for all methods.

	EFOAOA	EFO	AOA	MRFO	bGWO	HGSO	MPA	TLBO	SGA	WOA	SSA
D1	**2**	3	3	**2**	3	3	**2**	**2**	3	**2**	**2**
D2	**3**	14	5	4	**3**	5	4	4	18	4	10
D3	**2**	8	3	3	**2**	**2**	4	3	9	**2**	7
D4	**6**	7	**6**	**6**	8	7	**6**	**6**	8	8	7
D5	**3**	7	**3**	6	6	5	5	5	7	5	4
D6	**3**	7	5	4	7	7	5	**3**	9	7	5
D7	5	19	6	**2**	6	4	6	4	24	**2**	11
D8	9	24	12	13	17	11	10	11	27	**4**	20
D9	**2**	11	7	10	5	3	7	**2**	13	3	8
D10	**5**	8	6	6	7	7	6	6	8	8	7
D11	13	259	67	17	66	35	24	46	254	**10**	173
D12	21	45	17	13	15	16	21	17	45	**9**	28
D13	6	12	**4**	**4**	**4**	**4**	6	5	14	5	8
D14	5	5	5	6	**3**	4	6	5	5	**3**	5
D15	**2**	8	4	4	3	3	3	4	9	3	5
D16	22	28	9	17	11	**7**	13	10	32	12	21
D17	7	5	4	**3**	**3**	**3**	4	4	8	**3**	5
D18	4	4	**3**	4	6	5	**3**	4	7	6	5

**Table 7:** Accuracy results for all methods.

	EFOAOA	EFO	AOA	MRFO	bGWO	HGSO	MPA	TLBO	SGA	WOA	SSA
D1	0.9786	0.9629	0.9471	0.9619	0.9567	0.9752	0.9557	0.9729	0.9462	0.9476	**0.9790**
D2	**0.9899**	0.9684	0.9825	0.9760	0.9415	0.9333	0.9807	0.9573	0.9351	0.9433	0.9719
D3	**1.0000**	0.9609	0.9977	0.9716	0.9157	0.9854	0.9923	0.9655	0.9609	0.9448	0.9594
D4	**1.0000**	0.9820	**1.0000**	0.9993	0.8987	0.9743	0.9990	**1.0000**	0.8667	0.8960	0.9587
D5	0.7850	0.7270	0.7620	0.7650	**0.7900**	0.7260	0.7437	0.7507	0.7130	0.7703	0.7653
D6	**0.9089**	0.8889	0.8926	0.8654	0.8272	0.8914	0.9049	0.8877	0.8753	0.7988	0.8975
D7	0.9577	0.9127	0.9831	0.9681	0.9380	0.9117	0.9408	**0.9859**	0.9577	0.9174	0.9700
D8	**0.9781**	0.9713	0.9734	0.9779	0.9577	0.9495	0.9720	0.9547	0.9616	0.9507	0.9655
D9	**0.9685**	0.8299	0.9657	0.9289	0.8756	0.9319	0.9542	0.9337	0.8844	0.8821	0.9685
D10	**1.0000**	0.9820	**1.0000**	0.9990	0.9627	0.9853	**1.0000**	0.9990	0.9477	0.9497	0.9637
D11	**1.0000**	0.8667	0.8454	**1.0000**	0.9822	**1.0000**	0.9378	0.9511	0.8667	0.9686	**1.0000**
D12	0.9762	0.9381	0.9381	0.9571	0.9381	0.9556	0.9683	0.9794	**0.9937**	0.9825	0.9190
D13	0.8889	0.8111	0.8704	0.8506	0.7716	**0.9148**	0.8790	0.8531	0.8580	0.7593	0.8099
D14	**0.8666**	0.8052	0.8333	0.8226	0.7819	0.8101	0.8556	0.8354	0.8250	0.7809	0.7990
D15	0.9833	0.9467	0.9700	**0.9978**	0.9700	0.9844	0.9622	0.9711	0.9600	0.9622	0.9567
D16	**0.7710**	0.7394	0.7348	0.7665	0.7186	0.7283	0.7589	0.7530	0.7533	0.7283	0.7381
D17	**1.0000**	0.9833	**1.0000**	**1.0000**	0.9833	0.9981	**1.0000**	**1.0000**	0.9833	0.9759	**1.0000**
D18	**1.0000**	**1.0000**	**1.0000**	**1.0000**	0.9841	**1.0000**	**1.0000**	**1.0000**	**1.0000**	0.9968	**1.0000**

**Table 8:** Mean rank obtained using Friedman test for FS methods.

	EFOAOA	EFO	AOA	MRFO	bGWO	HGSO	MPA	TLBO	SGA	WOA	SSA
Accuracy	9.722	4.333	7.222	7.361	3.528	5.611	7.389	7.056	4.361	3.278	6.139
NO. Selected features	3.944	9.222	5.222	4.916	5.583	4.861	5.333	4.500	10.416	4.444	7.555
Fitness value	3.388	9.277	5.333	7.888	4.833	3.222	4.166	3.555	9.888	7.3889	7.055

**Table 9:** Comparsion with other existing FS methods.

Datasets	ECSA	EFOAOA	WOAT	WOAR	BBA	EGWO	BGOA	PSO	BBO	AGWO	SBO	bGWO2
D1	0.972	**0.978**	0.959	0.957	0.937	0.961	0.969	0.967	0.962	0.960	0.967	0.975
D2	0.958	**0.989**	0.949	0.950	0.931	0.947	0.96	0.933	0.945	0.934	0.942	0.935
D3	0.966	**1**	0.914	0.910	0.872	0.943	0.953	0.688	0.936	0.935	0.950	0.776
D4	**1**	**1**	0.739	0.763	0.61	0.753	0.946	0.73	0.754	0.757	0.734	0.75
D5	0.767	0.785	0.699	0.690	0.628	0.698	0.76	**0.787**	0.692	0.695	0.709	0.776
D6	0.83	**0.908**	0.765	0.763	0.754	0.761	0.826	0.744	0.782	0.797	0.792	0.7
D7	0.931	0.9577	0.884	0.880	0.877	0.863	0.883	0.921	0.880	0.893	0.898	**0.963**
D9	0.865	**0.968**	0.896	0.901	0.701	0.766	0.815	0.584	0.80	0.791	0.818	0.584
D10	**1**	**1**	0.778	0.759	0.722	0.870	0.979	0.737	0.880	0.878	0.863	0.729
D11	0.921	**1**	0.838	0.860	0.795	0.756	0.861	0.822	0.816	0.854	0.843	0.822
D12	0.926	**0.976**	0.736	0.712	0.844	0.861	0.895	0.928	0.871	0.882	0.894	0.938
D13	0.847	**0.888**	0.861	0.857	0.8	0.804	0.803	0.819	0.798	0.813	0.798	0.834
D14	0.842	0.866	0.792	0.778	0.665	0.771	**0.951**	0.735	0.768	0.762	0.768	0.727
D15	0.96	**0.983**	0.736	0.739	0.851	0.902	0.729	0.904	0.917	0.92	0.934	0.92
D17	0.985	**1**	0.935	0.932	0.919	0.966	0.979	0.933	0.966	0.957	0.968	0.92
D18	0.983	**1**	0.710	0.712	0.874	0.968	0.99	0.861	0.937	0.968	0.968	0.879

## Data Availability

The data are available from https://archive.ics.uci.edu/ml/index.php.
